# Female presence does not increase testosterone but still ameliorates sickness behaviours in male Japanese quail

**DOI:** 10.1098/rsos.220450

**Published:** 2022-05-24

**Authors:** Brenna M. G. Gormally, Kaelyn Bridgette, Aubrey Emmi, Delilah Schuerman, Patricia C. Lopes

**Affiliations:** Department of Biology, Chapman University, Orange, 1 University Drive, CA 92866, USA

**Keywords:** sickness behaviour, lipopolysaccharide, social interaction, testosterone, endotoxin

## Abstract

Infections can dramatically modify animal behaviour. The extent of these changes depends on an animal's environment. It has been proposed that testosterone modulates the suppression of behavioural symptoms of sickness under certain reproductive contexts. To further understand the role played by testosterone in modulating sickness behaviours under reproductive contexts, we studied a species, the Japanese quail, in which female exposure rapidly decreases circulating testosterone in males. Males received either an immune challenge (lipopolysaccharide – LPS) or a control injection and their behaviours, mass change and testosterone levels were quantified in the presence or absence of a female. Both the presence of a female and LPS treatment reduced testosterone levels. LPS-treated males maintained in isolation expressed expected sickness behaviours, including increased resting (quantified as crouching) and decreased food and water intake. Despite the reduction in testosterone, when paired with females LPS-treated males showed similar amounts of mating behaviours to controls and reduced crouching. In sum, even under very low levels of testosterone, male quail had reduced sickness behaviours when exposed to females, indicating that testosterone may not be key in modulating sickness behaviours, at least in this species.

## 1. Introduction

Infected animals undergo a suite of physiological and behavioural changes that are thought to help fight the infection [1]. The initial physiological wave of the immune response is characterized by the production of proteins and cytokines that activate and modulate the host's immune response (reviewed in [2,3]). These cytokines also help facilitate the production of ‘sickness behaviours’, consisting of lethargy, reduced food and water intake and altered social behaviours (reviewed in Lopes *et al*. [4]). Interestingly, the expression of sickness behaviours is modulated by both social and other environmental factors.

An important social modulator of sickness behaviours in males can be reproductive context [5]. For instance, immune-challenged male zebra finches (*Taeniopygia guttata*) show reduced sickness behaviours when in the presence of novel females [6], and when housed in groups [7].

Because behaviour is a major driver of disease spread [8,9], elucidating the mechanisms that explain variation in behavioural responses to infections is of critical importance to predict spread. However, the mechanisms behind the suppression of sickness behaviours remain incompletely understood. Some studies suggest testosterone may play a role. For example, seasonal differences in behavioural responses to an immune challenge were observed in free-living song sparrows (*Melospiza melodia morphna*), with stronger sickness behaviours occurring during the non-breeding season when testosterone titers are lower [10]. Immune-challenged gonadectomized Gambel's white-crowned sparrows (*Zonotrichia leucophrys gambelii*) that had received a testosterone implant presented reduced sickness behaviours relative to immune-challenged males that had not received exogenous testosterone [11]. By contrast to these observations, in Siberian hamsters (*Phodopus sungorus*) kept under long-days (when these animals have developed gonads and are producing testosterone), sickness behaviours (such as anorexia) of non-castrated (intact) males were enhanced upon an immune challenge, relative to those of castrated males [12]. What is interesting about this study in hamsters is that sickness responses (including sickness behaviours) were also changed by the photoperiod even in castrated animals, indicating that gonadal hormones, such as testosterone, do not paint the complete picture regarding the modulation of sickness behaviours. Therefore, the role of testosterone in modulating sickness behaviours remains unresolved.

In our study, we characterized sickness behaviours (lethargy, food and water intake, and crowing) in male Japanese quail (*Coturnix japonica*) when alone and tested the effect of a social stimulus, a novel female, on these and sexual behaviours. We selected Japanese quail for this study because, in contrast to many other bird species [13], male Japanese quail rapidly reduce (within 5–10 min) testosterone (Cornil *et al*. [14]; Delville *et al*. [14]) and luteinizing hormone (within 1 h; [15]) when presented with novel females. If testosterone indeed plays a role in suppressing sickness behaviours under reproductive contexts, then reducing testosterone concentrations (such as in response to a novel female) should prevent the reduction of sickness behaviours. This set-up allowed us to study the modulatory effect of female presence on sickness behaviours in a species where testosterone is reduced (rather than increased) by female presence.

## 2. Material and methods

Animals were kept on a 14 L : 10 D light cycle and at an average of 21°C. A total of 23 males and 30 females were used. Animals were hatched from fertilized eggs and reared in our laboratory as described in [16]. At approximately 6–8 weeks, same sex pairs were housed in chicken wire-divided cages (50 × 40 × 50 cm). Prior to running the experiments, males were given sexual experience with a female for at least 3 h. All males copulated during that period. Birds were 91 ± 16.6 days of age at the start of the experiment.

To elicit an immune response, lipopolysaccharides (LPS) were used. LPS is a component of the outer membrane of most Gram-negative bacteria and elicits an inflammatory response without exposing animals to replicating pathogens [4]. Males were randomly divided into two social treatments: males that either received a novel female (*n* = 15) or that remained isolated (*n* = 8). Males in both social treatments (female or isolated) were tested on two separate days, separated by 2.87 ± 1.46 days. On experimental day 1, males received a saline injection, and on experimental day 2, an LPS injection. With this design, males are their own controls for the effect of LPS. LPS was always given on the second experimental day because once inoculated, animals may show a placebo reaction to a second injection (of any kind), and because the effects of LPS on certain physiological systems may be longer lasting than anticipated (e.g. Brecchia *et al*. [17]; Valero *et al*. [18]), potentially affecting the outcome of our experiments.

During an experimental day, the male was weighed and injected in the pectoral muscle with either saline (endotoxin-free sterile PBS) or LPS (from *E. coli*; 2 mg k^−1^g; Sigma L4005 in.endotoxin-free sterile PBS) [19]. While LPS can be administered via different routes, research personnel were more easily trained on intramuscular injections, making it more consistent and safer for the birds, and our approved protocol, therefore, called for them. The quail were then placed alone in separate cages that were identical to their home cages and were monitored via security cameras (Axis M1065 L network camera, Axis Communications) throughout the experiment. At this time the quail were visually, but not audibly, separated from other quail.

At 30 min post injection, a sham injection in which the base of the syringe (no needle attached) was pressed against the pectoralis muscle was applied to all males, as a control for a separate experiment. At 40 min post injection, a blood sample (approx. 60 µl) was taken from the brachial vein of all animals, to also be used as part of a separate experiment.

At 1 h after injection (saline or LPS), males in the female treatment received one female in their cage. To control for the disturbance of adding a female, males in the isolation treatment had an experimenter enter the room, open the cage and insert their gloved hand in the cage. We allowed the male and female to spend 3 h together, at the end of which time we weighed the males once again and a blood sample (approx. 60 µl) was taken from the brachial vein. Blood samples were stored on ice until separation by centrifugation. Plasma was aspirated off of each sample and red blood cells and plasma were stored separately at −80°C until further analysis. Plasma samples were assayed for testosterone together using a previously validated [20] enzyme immunoassay kit (Item #582701, Cayman Chemical, Ann Arbor, MI, USA). Briefly, 5 µl of each plasma sample was diluted with 200 µl of UltraPure water and placed at 4°C for 30 min. At this point, 1 ml of diethyl ether was thoroughly mixed into each sample via a 10 s vortex; the sample was then left to separate for 20 min. A dry ice/ethanol bath was used to snap freeze the mixture and the top layer was poured off into a clean tube. This process was repeated twice more (total of three extractions), and the extracts were combined and left to evaporate in a 37°C water bath with nitrogen flowing over the tubes. Extracts were reconstituted with 400 µl of assay buffer (1 : 80 dilution). The manufacturer's instructions were followed to perform the assay. Samples were run in duplicate. Samples from this study were run alongside samples from additional studies, using a total of five plates. A plasma pool was run alongside samples on each plate; the inter-assay CV for this pool was 18.7%. Separate standard curves were run on each plate; the inter-assay CV of the standard curve was 11.1%. The intra-assay CV was 3.8% and assay sensitivity averaged 0.0084 ng ml^−1^ (calculated as the testosterone concentration at 80% bound). Five samples (all from LPS-treated birds) fell below the limit of detection and were assigned values based on the sensitivity of that plate.

Videos were analysed by trained observers who were blinded to treatment, using BORIS (BORIS [21]). Each observer coded all videos for a given bird, therefore covering both days of injections (saline and LPS). We quantified time spent crouching and eating, drinking and crowing bouts. Since sickness behaviours are characterized by lethargy, along with reduced eating and drinking, increases in crouching or reductions of eating, drinking and crowing would be indicative of sickness behaviours. Time spent attempting to mount and the number of cloacal contact movements (CCMs) [22] were quantified for males exposed to females. Behaviour was coded for 20 min after introduction of the female, as we expected the most dramatic behavioural responses during this initial period [14,23].

Crouching was defined as laying down still for more than three contiguous seconds. For crouching to be counted, the bird's abdomen had to be horizontal and in contact with the bottom of the cage. Mount attempts were classified as the time the male was attempting to mount or successfully mounting the female; this included neck grabbing [22]. Eating and drinking bouts began when the bird began pecking into the food dish or water bottle and ended when they walked away. Finally, each individual audible crow was counted.

Statistical analysis was performed in R v. 4.1.2 [24]. Box and whiskers plots were prepared using ggplot2 [25] and represent the median (central bold line), the first and third quartiles (lower and upper hinges, respectively), and the lowest and largest values no larger than 1.5 times the inter-quartile range (lower and upper whiskers, respectively). The raw values are also represented in those graphs, as circles.

The effect of treatments on testosterone and change in body mass were analysed using a repeated measures ANOVA that included the terms injection treatment, social treatment, an interaction of the two, and an error term consisting of bird identity. Because there was high inter-assay variation between plates of testosterone, plate number was initially added as a random effect to the testosterone model, but since it did not change the outcome of the analysis, it was dropped to simplify the model. Change in body mass was calculated as the difference between the mass at the end of a trial and the mass at the start of the trial. Data from none of the behaviours were normally distributed. To simplify the modelling for the effect of injection, for each animal, we calculated the difference between their behaviour when injected with LPS from their behaviour when injected with saline. We then applied a Wilcoxon rank-sum test (a non-parametric version of a one-way ANOVA), modelled as change in behaviour as function of social treatment. While these analyses were performed on deltas (LPS – saline), the box and whisker plots presented in the figures are of the raw data for clarity. Given that time spent attempting to mount and number of CCMs were only quantified in males paired with females, social treatment was not modelled. These two behaviours were analysed using a paired (to account for males being their own controls for the effect of injection) Wilcoxon signed-rank exact test (a non-parametric version of a one-way repeated measures ANOVA), modelled as behavioural response as a function of injection treatment.

## 3. Results

**Figure 1.**
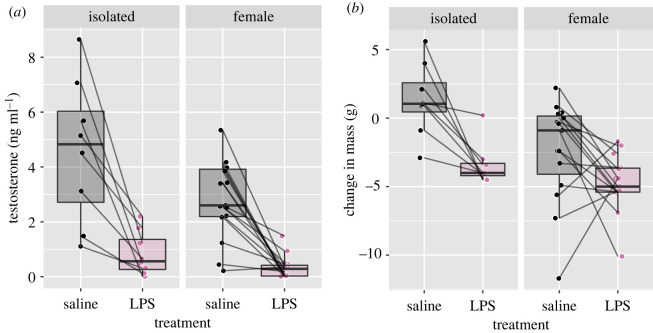
The effect of female presence and LPS treatments on testosterone and body mass. Both testosterone and change in body mass were affected by LPS treatment and by female presence (*p* < 0.05).

Both the presence of a female (*F*_1,21_ = 6.397, *p* = 0.0195) and LPS treatment (*F*_1,21_ = 52.991, *p* < 0.0001) reduced circulating levels of testosterone (figure 1*a*), but no interaction between these treatments was found (*F*_1,21_ = 2.307, *p* = 0.144). Similarly, both the presence of a female (*F*_1,21_ = 9.446, *p* = 0.00577) and LPS (*F*_1,21_ = 15.031, *p* = 0.000871) led to an overall reduction in body mass (figure 1*b*). There was no interaction between the treatments (*F*_1,21_ = 1.863, *p* = 0.186).

**Figure 2.**
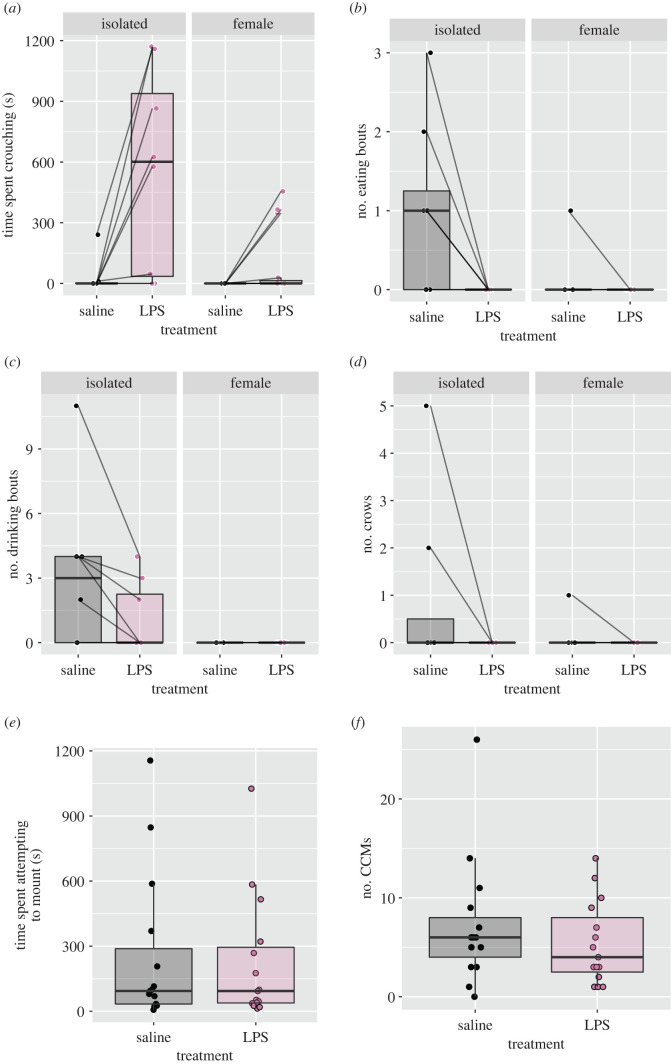
The effect of female presence and LPS treatments on behaviours. Social treatment affected the response to LPS for crouching, eating and drinking (*p* < 0.05), but not crowing. Mounting and CCMs were not different between saline and LPS treated males exposed to females (*p* > 0.05).

Social treatment affected time spent crouching in response to LPS (*W* = 98, *p* = 0.0075). In the isolation treatment, LPS increased time spent crouching, while in the female treatment, LPS did not elicit this increase in crouching (figure 2*a*). Social treatment also affected both the change in eating (*W* = 25.5, *p* = 0.0043) and drinking (*W* = 22.5, *p* = 0.00093) bouts in response to LPS. Eating and drinking were reduced by LPS injection in the isolation treatment, but not in the female treatment (figure 2*b*,*c*). This lack of effect of LPS when males were exposed to females appears driven by a reduction in eating and drinking behaviour by the saline males when exposed to females. Change in crowing in response to LPS was not significantly affected by social treatment (*W* = 48, *p* = 0.205; figure 2*d*). In terms of sexual behaviours (figure 2*e*,*f*), males in both the LPS and saline treatments spent similar amounts of time attempting to mount the female (*V* = 57, *p* = 0.89) and performed similar amounts of CCMs (*V* = 70.5, *p* = 0.27).

## 4. Discussion

Because testosterone has been proposed as a potential modulator of sickness behaviours in male birds [11], the motivation for this study was to understand whether animals that naturally reduce testosterone when exposed to a female can still modulate their sickness behaviours under this setting. We found that female presence was able to reduce male sickness behaviours even at extremely low testosterone levels. These data indicate that circulating testosterone may not be a key modulator of sickness behaviours, at least in Japanese quail.

Previous studies in Japanese quail have found that female presence rapidly reduces testosterone [14,23], but, to our knowledge, no studies have examined how this response might be impacted by immune challenges. Our results confirm that female presence reduces plasma testosterone and show that this effect is not modified by an immune challenge. As far as we are aware, a reduction of circulating testosterone following LPS treatment under reproductive contexts has not been previously described. One study in zebra finches found a trend for reduced testosterone after LPS treatment in a mixed-sex setting, where reproductive opportunities were available, but this effect was not significant [26]. A separate study in zebra finches, a species where female presence reduces sickness behaviours, found that LPS treatment tended to reduce testosterone in males that remained isolated, but not in animals that were paired with novel females [6]. The impact of LPS on the reproductive system may therefore be dependent on social context, an effect that would be interesting to examine further in future studies.

What mechanisms may underlie the social modulation of sickness behaviours? While systemic levels of testosterone may not be critical for the modulation of sickness behaviours, at least in Japanese quail, it is possible that neurosteroid production (i.e. production of steroids in the brain) could still have a role in modulating sickness behaviours. For example, in zebra finches, the expression of neuronal aromatase (an enzyme that converts testosterone to estradiol) increases after a peripheral injection with LPS [27]. Furthermore, if this aromatase production is inhibited, neuroinflammation is prolonged [28]. Given that neural estradiol affects both behaviours [29] and neuroinflammation, it is possible that the levels of neurosteroids in specific brain regions are more critical to the modulation of sickness behaviours than are peripheral levels. A different possible set of modulators would be monoamines, such as norepinephrine, dopamine, and serotonin. Monoaminergic activity in the brain can be rapidly changed by social stimuli [15,30–35]. Central monoamine levels are usually decreased by immune challenges [36–38]. The way in which different social stimuli alter central monoamines may therefore counteract some of the monoaminergic effects imposed by immune challenges, which could have implications for the behavioural output [39–42].

LPS treatment led to sickness behaviours (reduced overall activity) and increased mass loss, which is in line with previous studies that have used immune challenges to induce sickness behaviours in avian species and other vertebrates [4]. Mating behaviours were not reduced by an LPS injection, a clear demonstration of the social modulation of sickness behaviours. Female presence also was sufficient to reduce crouching time in LPS-treated animals. Interestingly, female presence reduced drinking and eating in saline males, to levels comparable to those seen in LPS-treated males. The presence of a female, therefore, seemed to equalize behavioural responses, likely because males dedicated a considerable amount of time and effort into mating behaviours once the females were around, regardless of being treated with LPS. Different social contexts that lead to reduced sickness behaviours go beyond mating context and include aspects such as maintenance of social status, territorial competition or offspring care [5]. It is possible that other social stimuli reduce sickness behaviours in this species, but we focused specifically on the presence of a female because this stimulus is known to quickly decrease circulating testosterone.

This modulation of sickness behaviours when in the presence of a female is consistent with previous studies that suggest animals will prioritize evolutionarily advantageous opportunities, even during an immune challenge [5]. However, contrary to previous observations [10,11], in our study this modulation occurred under a background of low testosterone levels, which suggests that testosterone may not always be essential for the social modulation of sickness behaviours.

In sum, our results provide new evidence that suggests testosterone may not be necessary as a modulator of sickness behaviours and that alternative mechanisms must enable males to suppress sickness behaviours in the presence of females. The mechanistic underpinnings of the social modulation of sickness behaviours remain a key question for future studies.

## Data Availability

Data and code are publicly available on https://doi.org/10.6084/m9.figshare.14949522.
